# Opportunities for recovery at work and excellent work ability - a cross-sectional population study among young workers

**DOI:** 10.1186/s12889-016-3654-0

**Published:** 2016-09-15

**Authors:** Maria Boström, Judith K. Sluiter, Mats Hagberg, Anna Grimby-Ekman

**Affiliations:** 1 Department of Occupational & Environmental Medicine, Sahlgrenska University Hospital, Postal: Box 414, SE 405 30 Gothenburg, Sweden; 2 Academic Medical Center, Department: Coronel Institute of Occupational Health, PO Box 22700, 1100 DE Amsterdam, The Netherlands; 3 Health Metrics, Sahlgrenska Academy, University of Gothenburg, Gothenburg, Sweden

**Keywords:** Varied work, Work ability score, Young workforce, Worktime control, Work-health promotion

## Abstract

**Background:**

Better opportunities for recovery at work are thought to be associated with work ability in a young workforce but evidence is scarce to lacking. The aim of this study was to examine cross-sectional associations between opportunities for recovery at work and excellent work ability among young workers and specifically for young workers with high work demands.

**Methods:**

A study group of 1295 women and 1056 men aged 18–29 years was selected from three biennial years of a population cohort. The subsample reporting high work demands consisted of 439 women and 349 men. The study group had completed a work environment questionnaire in a survey conducted by Statistics Sweden. Associations between opportunities for recovery at work and excellent work ability were assessed by multiple logistic regression models stratified for gender.

**Results:**

Having varied work was associated with excellent work ability in all young men (*p* < 0.0006; prevalence ratio [PR] 1.3) and also specifically in men with high work demands (*p* = 0.019; PR 1.3). For the latter group the possibility of deciding when to perform a work task was also associated with excellent work ability (*p* = 0.049; PR 1.3). Among young women with high work demands, the possibility of deciding one’s working hours was associated with excellent work ability (*p* = 0.046; PR 1.2).

**Conclusions:**

For young men, having varied work can contribute to excellent work ability. In addition, for men with high work demands, the possibility of deciding when to perform a work task may be favourable for excellent work ability. For young women with high work demands, the possibility of deciding one’s working hours can contribute to excellent work ability. Employers could use these opportunities for recovery in promoting work ability among young workers.

## Background

Young adults need good work ability to sustain them through a long working life as they replace the aging population [1]. Opportunities for recovery at work may be very important to this group as work environments for young workers, especially young women, are reported to be declining [2]. To our knowledge, however, associations between recovery opportunities at work and work ability have not previously been studied among young workers.

The definition of *work ability* can vary in different fields. In occupational health it is commonly defined as the balance between individual resources (e.g., health, knowledge, and attitudes) and working conditions (e.g., content, demands, and organization) [1]. In the present study work ability was measured by the work ability score (WAS) assessed in the work ability index (WAI) [3].

Several definitions for the concept of *recovery* include situational characteristics that diminish load effects [4], a need to recuperate from work-induced fatigue experienced primarily after a day of work [5], and a desire to be temporary relieved from demands in order to restore one’s resources [6]. As the last two definitions are related more to personal needs for immediate recovery, than to recovery opportunities afforded by structural aspects of work, the first definition was used in the current study.

Flexible working conditions have been found to offer recovery opportunities associated with health and well-being [7]. Worktime control has also been shown to be a promising tool to maintain health, well-being, and job-related outcomes, including performance [8]. The possibility of taking breaks at work and influencing other aspects of one’s working hours has been shown to have positive effects on work-related fatigue, sleep, and health complaints, but not on future absenteeism [4]. Rest breaks were shown in one review to have a positive effect on performance and productivity [9], while another review found that flexible and compressed workweek schedules also offered recovery opportunities that had a positive effect on performance and productivity [10]. Although several recovery opportunities at work have been studied and seem to have an effect on health, well-being, performance, and productivity, these have not been studied specifically in young workers.

Though no studies of associations between recovery opportunities at work and work ability have been found for young workers, there are some closely related studies. In an interview study on work ability, recovery opportunities at work were included in a nuanced picture of experiences of work ability among young workers [11]. Further, young women have been found to strive for balance between stress and recovery at work to maintain their health and work ability [12]. One interesting hypothesis suggests that lack of recovery associated with prolonged work schedules and overtime can generate pressure on work ability among young workers [13], but specific recovery opportunities at work are not discussed.

Because young workers (aged 16–29 years), especially young women, report more strenuous work and experience more fatigue after work than older workers [2], recovery opportunities during the working day could be important in promoting excellent work ability. According to this report by the Swedish Work Environment Authority, young workers had less opportunity to decide when to do a work task and had more monotonous work than older workers; consequently, the work environment of young Swedish workers is poorer and includes fewer recovery opportunities that that of workers aged 30 years and older. Young workers with high work demands most likely have a greater need of recovery at work and hence a work environment with recovery opportunities than those with fewer demands [14].

The concept of recovery is complex. It includes both recovery during the work day (internal) and recovery between work days (external) [14]. While we recognize this complexity, the present study focused solely on recovery opportunities at work since this topic appears to be unexplored among young workers.

The aim of this study was to investigate the association between opportunities for recovery at work and excellent work ability among young workers, especially for young workers with high work demands. Specific research questions were: i) is the probability higher to report excellent work ability when a higher degree of recovery opportunities at work are present?, ii) does the association in i) for the total sample hold when adjusted for high work demands and educational level? and iii) is the association between recovery opportunities at work and excellent work ability stronger among those with high work demands?

## Methods

### Study design and data collection

This is a cross-sectional population study using the Work Environment Survey by Statistics Sweden on behalf of the Swedish Work Environment Authority. This survey is based on the Labour Force Survey and includes additional questions asked in a telephone interview and a follow-up postal questionnaire. The purpose of the survey is to describe the physical and psychosocial work environment of the employed population. The employed population was defined as those aged 16–64 years who worked at least one hour during the measurement week. The Work Environment Survey has been conducted every second year since 1989. For each of the years 2009, 2011, and 2013 the randomized selected sample was about 10 000–16 000, of whom approximately 8100– 12 400 answered the telephone interview and about 4800–7800 also answered the postal questionnaire. The telephone interview asks participants background questions on employment, work strain, leadership, and work ability. A week later the postal questionnaire asks about 121 specific occupational and health items. The development and validation of the method is described in a report from Statistics Sweden [15].

### Study sample

**Fig. 1:**
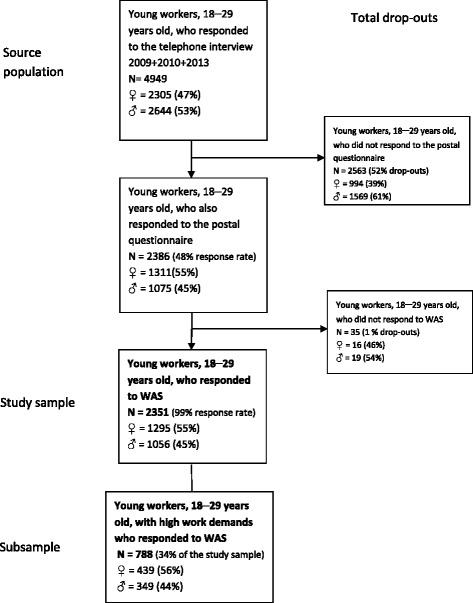
Selection of the study group. The study group included young workers who answered the telephone interview, the questionnaire, and also the question about work ability ranked according to Work Ability Score, WAS

In 2009–2013 about 19 000 individuals responded to the full telephone interview. Of these 4949 were young workers, 18–29 years of age. The study sample consists of 2351 young workers who responded to the telephone interview, the postal questionnaire, and the WAS question in the telephone interview (Fig. 1). There was no overlap of individuals in the three years chosen for the study. Inclusion criteria for the study sample were age range 18–29 years, and having answered the single WAS question. For the subsample of individuals with high work demands, *N* = 788, the criterion was to have reported having either a job that was generally physically strenuous to a large extent *or* a high workload with far too much to do to a large extent, *or* both. The selection of this subsample was chosen as women and men with high work demands could have an increased need of recovery opportunities at work [14].

### Individual characteristics

Individual characteristics drawn from the survey were used to describe the sample: sex, age, education, years in present occupation, trade, work ability, and health. The variable educational level was dichotomized into compulsory school or high school versus post-gymnasium, college, or university. Three health variables were identified: pain in at least one location including upper back or neck, lower back, shoulder or arms, wrists or hands, and hips, legs, knees or feet; feeling very tired after work; and sleep difficulties due to thoughts of work. These health variables were ranked by frequency: every day, a few days per week, a day per week, a few days per month, seldom or not at all. The three first alternatives implied impaired health.

### Outcome

#### Excellent work ability

Work ability can be measured by using part of the work ability index (WAI), a Finnish self-report instrument with seven dimensions measured on 10 items, by which individuals assess their own work ability [1]. In the present survey work ability was measured by using the WAS [3], taken from, and shown to correspond with, the WAI [16, 17]. The WAS measures “current work ability compared with the lifetime best” on a scale ranging from 0 “cannot work at all right now” to 10 “my work ability is at its best right now”. Excellent work ability was ranked 10 on the scale, as in other studies [3, 16].

### Explanatory variables

#### Recovery opportunities at work

Because of the lack of knowledge of possible associations between recovery opportunities at work and excellent work ability, the five items measuring recovery opportunities at work in this study were chosen mainly from research about associations between recovery opportunities at work and health, well-being, performance, and productivity. Factors in accord with the Recovery Opportunity Scale [4], which measures recovery opportunities in relation to health, were also used. Overall, the items in the questionnaire were used to assess opportunities for recovery at work, not lack of recovery at work.

The first question, on the possibility of deciding one’s own working hours [4, 10, 18], had three answer alternatives: “Yes, I can be on flexitime”, “Yes, I have in other ways relatively free working hours”, and “No, I cannot influence my working hours”. The first two alternatives were considered recovery opportunities.

The second question asked about the possibility of deciding the work pace. Although this was not previously considered a recovery factor [19], it was included in the hypothesis because it occurs in regulations on workers’ opportunities to influence their own work [20]. A third question asked about the possibility of taking short breaks at any time to talk, [4, 9, 18]. These two questions had the same response alternatives:” nearly all the time” and “about three quarters of the time” represented recovery opportunities at work, in contrast to the alternatives of “half the time”, “about a quarter of the time”, “a little” and “no, not at all”.

The fourth question, about the possibility of deciding when to perform a work task [8], was answered by “always”, “mostly”, “mostly not”, or “never”, with the two first alternatives signifying recovery opportunities at work. The fifth and last question asked about variation in the respondents’ work. On a scale of 0–5, responses ran from “monotonous work” to “varied work”, and ratings of 4 and 5 indicated an opportunity for recovery at work. This question was chosen because the opportunity to take spontaneous breaks increases with varied work [14].

### Possible confounders

High work demand was seen as a possible confounder for the analyses of the total sample to answer the first research question. This variable was, however, also used for creating a subsample, as described in the study sample section, to answer the third research question. For the second research question, two possible confounders were considered: high work demands and educational level. High work demands can influence work ability [21] and have a negative association with recovery opportunities at work [14] and was defined in terms of physically strenuous work to a large extent *and/or* high workload with far too much to do to a large extent. Poor basic education has been shown to relate to poor work ability among young adults [13] and was therefore considered as a confounder.

### Statistical analysis

SAS version 7.11 (SAS Institute, Cary, NC, USA) was used for the analyses in this study. In all analyses the data were stratified for women and men [22]. A subsample of those with high work demands was also analyzed. Descriptive data were obtained through frequency analyses.

The associations between recovery opportunities at work and excellent work ability was analyzed with logistic regressions (proc genmod in SAS). The effect measures retrieved from these regressions were prevalence ratios (PRs) instead of the commonly used odd ratios (ORs). This is due to the outcome being common and hence ORs cannot be interpreted as approximations of PRs. From the logistic regressions PRs were calculated as the effect of the exposure around the prevalence of 50 %. In the presented PRs the group with the lower degree of recovery opportunities at work was used as the reference category. The 95 % confidence intervals (CIs) for PRs were calculated using the delta method [23].

A guideline of applied logistic regression was followed [24] to build models for testing associations between recovery opportunities at work and excellent work ability. Co-linearity was checked by examining pairwise correlations by cross tables considering > 80 % in a diagonal or any cells with no answers. This was performed between all exposure variables, between all exposure variables and the possible confounders of high work demands and educational level, and between the two confounders. *“Being involved in planning the work”* showed co-linearity with the variable *“Possibility of deciding when to perform a work task*”. *“Being involved in planning the work”* was therefore excluded from further analyses due to more missing compared to *“Possibility of deciding when to perform a work task*”. Univariate logistic regression analyses were then performed to select variables for the multiple regression with the criteria of *p* < 0.25.

The steps in the guideline for creating multiple models set criteria for variable inclusion at *p* < 0.25, changes in parameter estimates for exposure variables at < 15 %, and likelihood ratio tests between nested models. If an explanatory variable had *p* ≥ 0.25, but changed parameter estimates for other explanatory variables when the models were considered with and without it, it was included as a confounder. Possible interactions between exposure factors were also tested.

### Ethics

This study was approved by the Regional Ethical Review Board at the University of Gothenburg, Sweden (Reg.no. 221–15).

## Results

### Characteristics of the study sample

The proportion of women was 55 % in the study sample and 56 % in the subsample (Table 1). High work demands were reported by 34 % of the women (*N* = 439) and 33 % of the men (*N* = 349). Self-reported work ability was high for women and men (9.2) overall and in the group with high work demands (9.0–9.1). The young workers, especially women, generally had few years of work experience. The men seemed to have lower educational levels than the women, especially among those with high work demands.

**Table 1:** Characteristics of the study sample and the subsample

Young workers without/with high work demands	Women		Women with high work demands		Men		Men with high work demands	
N = 2351/N = 788	N = 1295 (55 %)		N = 439 (56 %)		N = 1056 (45 %)		N = 349 (44 %)	
*Individual factors*								
Work ability: mean	9.2		9.0		9.2		9.1	
range	1–10		1–10		1–10		1–10	
SD	1.3		1.4		1.2		1.4	
Age in years: mean	24.3		24.3		24.8		24.7	
range	18–29		18–29		18–29		18–29	
SD	3.3		3.2		3.2		3.1	
Years in present occupation: mean	2.5		2.8		3.1		3.7	
range	1/12–14		1/12–14		1/12–15		1/12–15	
SD	2.3		2.5		2.5		2.8	
	N	%	N	%	N	%	N	%
Excellent work ability	814	63	252	57	673	64	204	58
Educational level								
Compulsory school/high school	705	55	261	60	692	66	261	75
Post–gymnasium/college/university	574	45	173	40	356	34	85	25
Pain in at least one location of the body at least one day per week the last three months	791	62	318	75	452	44	198	58
Tired out after work at least one day per week the last three months	699	55	324	75	515	49	245	71
Sleep difficulties due to thoughts of the work keeping you awake at least one day per week the last three months	246	19	113	26	155	15	70	20
*Recovery opportunities*								
Possibility of deciding working hours								
Yes, flexible working hours or free working hours	742	58	208	48	616	59	164	47
No, cannot influence working hours	544	42	229	52	433	41	184	53
Possibility of deciding the work pace								
At least 3/4 of the time	484	38	114	26	495	47	145	42
At most half of the time	803	62	322	74	557	53	204	58
Possibility of taking short breaks								
At least 3/4 of the time	495	39	106	24	577	55	155	45
At most half of the time	790	61	330	76	466	45	189	55
Possibility of deciding when to perform a work task								
Mostly or always	581	45	152	35	604	58	172	50
Mostly not or never	701	55	280	65	445	42	174	50
Having work that is mostly								
Varied	548	43	172	40	411	39	126	36
Monotonous	740	57	263	60	635	61	222	64

(*N* number of workers, *SD* standard deviation)

The young women and men tended to work in different trades (not shown in the tables). The largest group of women, both in the whole sample and in the subsample with high work demands, was found in the service, health care, and retail sector. Men were most often employed in construction and manufacturing, and then in the service, health care, and retail sector, though men with high work demands worked mainly in construction and manufacturing.

Women with high work demands reported the poorest health (pain, fatigue, and sleep difficulties), although pain and fatigue were common in the whole sample (Table 1).

Recovery opportunities at work were reported at various levels, as about half of the study sample experienced some recovery at work. Those with high work demands, especially women, seemed to report fewer of several of the investigated recovery variables than the sample as whole. Possibilities of deciding one’s own working hours and to have varied work appeared to be quite similar for women and men in general, but at least the first variable seemed to be less common among those with high work demands.

### Associations between recovery opportunities at work and excellent work ability

Univariate associations between recovery opportunities at work and excellent work ability for both young women and men are presented in Table 2. For men only, *possibility of deciding the work pace*, *possibility of taking short breaks, possibility of deciding when to perform a work task,* and *varied work* showed unadjusted associations with excellent work ability. For men with high work demands, all of these variables except for *possibility of taking short breaks* also showed unadjusted associations with excellent work ability.

**Table 2:** Univariate associations between recovery opportunities at work and excellent work ability

	Women				Women with high work demands			
			N = 1282–1289				N = 435–437	
	Exposed (n)	Cases (n)	Univariate model		Exposed (n)	Cases (n)	Univariate model	
*Recovery opportunities at work*			PR	(95 % CI)			PR	(95 % CI)
Possibility of deciding working hours	742	485	1.1	(0.998–1.26)	208	130	1.2	(0.98–1.44)
Possibility of deciding the work pace	484	316	1.1	(0.96–1.22)	114	73	1.2	(0.94–1.48)
Possibility of taking short breaks	495	322	1.1	(0.95–1.20)	106	63	1.1	(0.82–1.29)
Possibility of deciding when to perform a work task	581	374	1.1	(0.94–1.18)	152	92	1.1	(0.88–1.33)
Having mostly varied work	548	356	1.1	(0.95–1.20)	172	101	1.0	(0.83–1.24)
	Men				Men with high work demands			
			N = 1043–1053				N = 344–349	
	Exposed (n)	Cases (n)	Univariate model		Exposed (n)	Cases (n)	Univariate model	
*Recovery opportunities at work*			PR	(95 % CI)			PR	(95 % CI)
Possibility of deciding working hours	616	401	1.1	(0.93–1.21)	164	98	1.0	(0.82–1.27)
Possibility of deciding the work pace	495	343	1.2	(1.09–1.41)	145	96	1.3	(1.03–1.62)
Possibility of taking short breaks	577	394	1.2	(1.09–1.41)	155	99	1.3	(0.98–1.53)
Possibility of deciding when to perform a work task	604	414	1.3	(1.11–1.43)	172	114	1.4	(1.06–1.66)
Having mostly varied work	411	296	1.4	(1.17–1.54)	126	86	1.4	(1.06–1.71)

(*N* number of workers included in the univariate analyses, *n* number of workers exposed for the variable respectively being a case, *PR* prevalence ratios, 95 % CI = 95 % confidence interval)

The variables included in the multiple models are shown in Table 3. For women, the exposure variable *possibility of deciding working hours* was included in the multiple model I with *p* = 0.08. In the multiple model II the same exposure variable was included; however, *p* fell to 0.1. *Possibility of deciding the work pace* was included in both these models only to adjust the models and thus should be viewed as a confounder. In the multiple model III for women with high work demands, both *possibility of deciding working hours* and *possibility of deciding the work pace* were included, however, with *p* = 0.1 and *p* = 0.2, respectively. In the multiple model IV for women only *possibility of deciding working hours* was included with *p =* 0.046.

**Table 3:** Multiple regression model. Parameter estimates (b coefficient) and p (*p*-values) for associations between recovery opportunities at work and excellent work ability for the study sample and the subsample

	Women				Women with high work demands			
	N = 1279				N = 434			
	Multiple model I^a^		Multiple model II^b^		Multiple model III^c^		Multiple model IV^d^	
	Parameter estimates	P	Parameter estimates	P	Parameter estimates	P	Parameter estimates	P
*Intercept*	0.363	<0.0001	0.504	<0.0001	0.082	0.556	−0.086	0.644
*Recovery opportunities at work*								
Possibility of deciding working hours	0.212	0.077	0.180	0.137	0.320	0.109	0.395	**0.046** ^f^
Possibility of deciding the work pace	0.133	0.283^e^	0.090	0.472^e^	0.291	0.207	.	.
Possibility of taking short breaks	.	.	.	.	.	.	.	.
Possibility of deciding when to perform a work task	.	.	.	.	.	.	.	.
Having mostly varied work	.	.	.	.	.	.	.	.
*Confounder*								
High work demands	.	.	−0.306	0.013	.	.	.	**.**
Educational level	.	.	.	.	.	.	0.336	0.094
	Men				Men with high work demands			
	N = 1025				N = 345			
	Multiple model I^a^		Multiple model II^b^		Multiple model III^c^		Multiple model IV^d^	
	Parameter estimates	P	Parameter estimates	P	Parameter estimates	P	Parameter estimates	P
*Intercept*	0.043	0.715	0.162	0.223	−0.150	0.391	−0.416	0.139
*Recovery opportunities at work*								
Possibility of deciding working hours	.	.	.	.	.	.	.	.
Possibility of deciding the work pace	0.240	0.103	0.236	0.110	0.351	0.162	0.256	0.317^e^
Possibility of taking short breaks	0.197	0.173	0.166	0.256^e^	.	.	.	.
Possibility of deciding when to perform a work task	0.246	0.097	0.232	0.118	0.403	0.099	0.491	**0.049** ^f^
Having mostly varied work	0.488	**0.0005** ^f^	0.484	**0.0006** ^f^	0.526	**0.027** ^f^	0.579	**0.019** ^f^
*Confounder*								
High work demands	.	.	−0.268	0.056	.	.	.	.
Educational level	.	.	.	.	.	.	0.334	0.213

^a^Multiple model with no confounders. ^b^Multiple model with two confounders: high work demands and educational level. ^c^Multiple model for the subsample with no confounders. ^d^Multiple model for the subsample with one confounder: educational level. ^e^Variable included only to adjust the multiple model. ^f^The bold figures representing *p*-values < 0.05. (N = number of workers included in the multiple analyses, . = the variable was not included in the multiple model)

Model I for the study sample of women included neither of the specified confounders (high work demands and educational level), but model II for the study sample of women included high work demands, Table 3. Further, model III for the subsample of women did not include the educational confounder, but model IV for the subsample of women did.

Other exposure variables were included in the multiple models for men. Having reported *varied work* was included in the two multiple models for all men, I and II, and also for men with high work demands, models III and IV, all with *p* < 0.05. *Possibility of deciding when to perform a work task* was included in both models I and III, with *p* = 0.097 and *p* = 0.099, respectively. In model II, this exposure variable was included with *p* = 0.1, and in model IV, *p* for this variable was 0.049. *Possibility of deciding the work pace* was included in models I, II, and III, with *p* = 0.1, *p* = 0.1, and *p* = 0.2, respectively. Finally, the variable *possibility of taking short breaks* was included, with *p* = 0.2 for all men in model I. In model II this exposure variable was only included to adjust the multiple model, as was *possibility of deciding the work pace* in model IV, and these should therefore be seen as confounders.

Similar to women, shown in Table 3, model I for the study sample of men included neither of the specified confounders (high work demands and educational level), and model II for the study sample of men included high work demands. Further, model III for the subsample of men did not include the educational confounder, but model IV for the subsample of men did, also in similarity with women.

The size of the effect of recovery opportunities at work on excellent work ability for young women is shown in Table 4 and for young men in Table 5. The largest effects were found for men who reported *varied work* (PR = 1.3 in all models), for men with high work demands with *possibility of deciding when to perform a work task* (PR = 1.3, model IV), and for women with high work demands who reported *possibility of deciding working hours* (PR = 1.2, models III and IV).

**Table 4:** Prevalence ratios. Prevalence ratios based on multiple regression models for associations between recovery opportunities at work and excellent work ability for young women

	Women				Women with high work demands			
	N = 1279				N = 434			
	Multiple model I^a^		Multiple model II^b^		Multiple model III^c^		Multiple model IV^d^	
*Recovery opportunities at work*	PR	(95 % CI)	PR	(95 % CI)	PR	(95 % CI)	PR	(95 % CI)
Possibility of deciding working hours	1.1	(0.98–1.24)	1.1	(0.96–1.22)	1.2	(0.94–1.41)	1.2	(0.98–1.46)
Possibility of deciding the work pace	1.1^e^	(0.94–1.20)	1.0^e^	(0.92–1.17)	1.2	(0.89–1.42)	.	.
Possibility of taking short breaks	.	.	.	.	.	.	.	.
Possibility of deciding when to perform a work task	.	.	.	.	.	.	.	.
Having mostly varied work	.	.	.	.	.	.	.	.

^a^Multiple model with no confounders. ^b^Multiple model with two confounders: high work demands and educational level. ^c^Multiple model for the subsample with no confounders. ^d^Multiple model for the subsample with one confounder: educational level. ^e^Variable included only to adjust the multiple model

(*N* number of workers included in the multiple analyses, *PR* prevalence ratios, 95 % CI = 95 % confidence interval, . = the variable was not included in the multiple model)

**Table 5:** Prevalence ratios. Prevalence ratios based on multiple regression models for associations between recovery opportunities at work and excellent work ability for young men

	Men				Men with high work demands			
	N = 1025				N = 345			
	Multiple model I^a^		Multiple model II^b^		Multiple model III^c^		Multiple model IV^d^	
*Recovery opportunities at work*	PR	(95 % CI)	PR	(95 % CI)	PR	(95 % CI)	PR	(95 % CI)
Possibility of deciding working hours	.	.	.	.	.	.	.	.
Possibility of deciding the work pace	1.1	(0.96–1.29)	1.1	(0.96–1.29)	1.2	(0.90–1.49)	1.1^e^	(0.85–1.42)
Possibility of taking short breaks	1.1	(0.95–1.26)	1.1^e^	(0.93–1.24)	.	.	.	.
Possibility of deciding when to perform a work task	1.1	(0.97–1.30)	1.1	(0.96–1.29)	1.2	(0.93–1.52)	1.3	(0.96–1.60)
Having mostly varied work	1.3	(1.10–1.46)	1.3	(1.10–1.45)	1.3	(0.99–1.61)	1.3	(1.01–1.67)

^a^Multiple model with no confounders. ^b^Multiple model with two confounders: high work demands and educational level. ^c^Multiple model for the subsample with no confounders. ^d^Multiple model for the subsample with one confounder: educational level. ^e^Variable included only to adjust the multiple model. (*N* number of workers included in the multiple analyses, *PR* prevalence ratios, 95 % CI = 95 % confidence interval, . = the variable was not included in the multiple model)

## Discussion

To have varied work was found to be a recovery opportunity associated with excellent work ability for young men. In addition, for men with high work demands, the possibility of deciding when to perform a work task was also associated with excellent work ability. For young women with high work demands, the recovery opportunity to decide one’s own working hours was associated with excellent work ability.

### Associations between recovery opportunities at work and excellent work ability

The most distinct finding in the current study was that young men with varied work seemed most likely to experience excellent work ability. The recovery obtained from varied work among the men can probably include both the possibility to take breaks, which has been shown to maintain performance among adult workers [9], and the possibility of deciding the work pace, both of which are in line with a theoretical framework of recovery in relation to variety in the job setting [14]. A review of studies in work physiology has clearly shown that variation in physical workload has a significant influence on recovery in relation to musculoskeletal disorders [25]. Creating varied work content could therefore contribute to promoting excellent work ability in young men, especially since younger workers tend to have more monotonous work than older workers [2].

The association between varied work and excellent work ability was not, however, any stronger for young men with high work demands. This indicates that varied work is important for excellent work ability, independent of the level of work demands. Further, the different results for varied work in women and men might be explained by gender segregation of different work tasks in the labour market [26], with different contributions to variation of work postures and work movements. Even in the same occupation it is known that work tasks often differ between women and men [27], and men are therefore more likely able to create variation in their work.

For men with high work demands, the possibility of deciding when to perform a work tasks was also found to be associated with excellent work ability. This recovery opportunity is included in the concept “global worktime control”, associated with job satisfaction, but not with performance or productivity [8]. Hence, the result of work ability in the current study has not, to our knowledge, been presented earlier.

Enabling women with high work demands to decide their own working hours could be a promising way to promote excellent work ability. This result is in line with earlier studies among adult workers. Having flextime has been shown to have an impact on job satisfaction [8] and a positive effect on productivity [10], although previously reported results were not specific for young women. Adult women with poor health and the opportunity to adjust their worktime were prospectively associated with increased work ability and return to work [28]; however, a large part of that study’s sample had poor work ability at baseline, which casts doubt on the appropriateness of a direct comparison with results of the current study. As no other recovery opportunities at work were found in the present study to be associated with excellent work ability among young women, other external factors such as duration and/or quality of sleep and/or relaxation between work-days may, in our opinion, be more important to their maintaining excellent work ability. Consequently, the situation could be more complex for young women, and possible important recovery opportunities were not taken into account in this study.

### Methodological considerations

Obvious strengths of this study include its large population sample of a group not earlier investigated, but it also has limitations. The high proportion of young workers reporting excellent work ability and reporting high work ability in general might have muted the contrasts between exposed and unexposed groups and at least in part account for the small effects that were found. Furthermore, the limited scope of taking only internal recovery into account could make interpretations of the results uncertain, but probably contributes to a clearer focus.

The possible cohort effects in the current study may be an additional weakness as the sample was selected from different years. This sampling method of using surveys from three subsequent years was selected to obtain large groups for a study sample stratified for gender and further divided into subsamples with high work demands, while retaining enough power in the statistical analyses. Although the general work environment did actually change over the six years of the study [2], there is no reason to assume that possible associations between recovery opportunities at work and excellent work ability should also have changed substantially.

Excellent work ability can be measured in different ways. In the present study, as in earlier studies [3, 16], the cutoff for excellent work ability was 10 on the WAS. However, scores of 9–10 on the WAS have also been used for young workers [13, 29], in circumstances where choosing a score of 10 would have resulted in too few cases. Because self-rated work ability was high in the study sample, 10 on the WAS was considered appropriate for excellent work ability.

The questions in the telephone interview and the questionnaire were validated by Statistics Sweden, as occupational demands can be difficult to assess by self-reported exposure [30]. The validation procedure for these questions has been carefully described [15], and the questions used in the self-reported questionnaire were found to give reliable descriptions of actual work environments and conditions. Following that validation study, further work to increase the validity and reliability of the questions was also undertaken by Statistics Sweden; however, this work has not been carried out to a large extent since 2009, so the questions set for the three cohorts in the present study were nearly identical in formulation.

The choice of age group in the study sample warrants discussion. The upper age limit of 29 follows the limit for young workers used by Statistics Sweden, although their group starts at 16 years old. The lower limit of 18 years was chosen because most young adults aged 16–18 years continue in high school, and we wanted to examine young people at work.

The main limitation of the present study is the cross-sectional study design, which hinders interpretation of possible causal associations. Despite this limitation, however, the study has some obvious strengths. The design of a population-based register study is a broad attempt to capture the topic among a large group of young employees, and the well-described method selected for building the multiple models can be seen as an advantage. Furthermore, the research topic is, to our knowledge, poorly investigated in young workers, despite its possible importance to a sustainable working life for this young group.

### Applications

The results of this study could be used when planning organizational actions to promote excellent work ability among young workers. Varied work might contribute to excellent work ability in men and plausibly a healthier workplace for all employees [31, 32]. Also, facilitating worktime control could be one way to promote excellent work ability, especially for women with high work demands, though the awareness of other opportunities for recovery at work and in leisure may be more important. Further studies, longitudinal or qualitative, are greatly needed to explore how recovery opportunities through the workplace can contribute to excellent work ability among young working women and men.

## Conclusions

For young men, having varied work can contribute to excellent work ability. In addition, for men with high work demands, the possibility of deciding when to perform a work task may be favourable for excellent work ability. For young women with high work demands, the possibility of deciding one’s working hours can contribute to excellent work ability. Employers could use these opportunities for recovery in promoting work ability among young workers.
